# Angiotensin-converting enzyme-2 overexpression improves atrial electrical remodeling through TRPM7 signaling pathway

**DOI:** 10.18632/oncotarget.20221

**Published:** 2017-08-12

**Authors:** Tingquan Zhou, Zhihua Han, Jun Gu, Shaojie Chen, Yuqi Fan, Huili Zhang, Yuehui Yin, Junfeng Zhang, Changqian Wang

**Affiliations:** ^1^ Department of Cardiology, Shanghai Ninth People’s Hospital, Shanghai JiaoTong University School of Medicine, Shanghai, China; ^2^ Department of Cardiology, Shanghai First People’s Hospital, Shanghai JiaoTong University School of Medicine, Shanghai, China; ^3^ Department of Cardiology, The Second Affiliated Hospital of Chongqing Medical University, Chongqing, China

**Keywords:** angiotensin II, angiotensin-converting enzyme 2, atrial fibrillation, atrial electrical remodeling, transient receptor potential melastatin-7

## Abstract

Atrial electrical remodeling is an important factor in the development and persistence of atrial fibrillation. The aim of this study was to examine the effects of atrial angiotensin-converting enzyme-2 overexpression on atrial electrical remodeling and to elucidate the molecular mechanisms underlying these effects. Twenty-eight male and female dogs were randomly divided into the following 4 groups: a sham-operation group, a control group, an adenovirus-enhanced green fluorescent protein (Ad-EGFP) gene group and an Ad-ACE2 gene group. All dogs in the Ad-EGFP and Ad-ACE2 groups were rhythmized at 450 bpm for 14 days. Two weeks later, all the dogs underwent thoracotomy and epicardial gene painting. On day 21 after gene transfer, all the animals were subjected to electrophysiological and molecular studies. AF induction rates and durations were significantly increased in the control and Ad-EGFP groups compared to the sham-operated and Ad-ACE2 groups. Transient receptor potential melastatin 7 (TRPM7) expression levels in the Ad-EGFP and control groups were significantly higher than those in the sham-operated and Ad-ACE2 groups.

Basal [Mg^2+^]_i_ was significantly decreased in siRNA transfected cells compared with control and non-silencing siRNA-transfected cells. Our results suggest that ACE2 overexpression suppresses atrial electrical remodeling and improves atrial function through the TRPM7 signaling pathway.

## INTRODUCTION

Atrial fibrillation (AF) is the most common arrhythmia and has a significant effect on overall mortality and morbidity [1]. The AF recurrence rate is still high despite therapy [2]. The main reason for the poor efficacy of AF therapy is atrial remodeling, in which the renin-angiotensin system (RAS) is involved [3]. Ang II is an important member of the RAS and is associated with the development of AF. Ang-(1-7), another important member of the RAS, is able to balance the effects of Ang II. Ang II is converted to Ang (1-7) by the ACE homologue Ang-converting enzyme (ACE) 2 [4]. The results of an Ace2 mutant mouse experiment showed that ACE2 negatively regulates RAS activity [5]. Therefore, ACE2 may play an important role in atrial electrical remodeling during AF. However, the detailed mechanism of action of ACE2 in atrial fibrillation has not been fully elucidated. In recent years, researchers have found that TRP channels play a role in many physiological and pathological processes. TRPM7 is the predominant Ca2+ channel in cardiac fibroblasts [6-8]. Therefore, the aim of this study was to determine whether ACE2 overexpression improves atrial electrical remodeling and function through the Ang II/AT1/TRPM7 pathway.

## RESULTS

### Changes in electrophysiological variables

The general characteristics of the experimental dogs are shown in Table 1. No differences in baseline characteristics were observed among the 4 groups. The two basic pericardial atrial effective refractory periods (AERPs) of the right and left atria of all the groups are shown in Table 2. Compared to the Sham group, the AERPs were significantly reduced in all the experimental groups at 3 weeks after gene transfer. Electrophysiological studies were performed to determine AF induction rates and durations. On the 35th day after gene transfer the AF induction rates of the control and Ad-EGFP groups were significantly increased compared to that of the Ad-ACE2 group. Additionally, on day 35, the AF durations of the control and Ad-EGFP groups were significantly increased compared to those of the sham-operated and Ad-ACE2 groups (Table 3).

**Table 1:** General condition of the experimental dogs

	Sham	Control	Ad-EGFP	Ad-ACE2	*P*
Male	4/7	4/7	3/7	3/7	0.903
Weight(Kg)	23.1±5.0	25.4±5.2	23.7±4.9	25.9±6.6	0.752
Heart rate(beat/min)	149.3±15.7	145.6±20.6	145.9±19.9	143.7±21.5	0.96
White blood cell(*10^9^)	7.8±1.2	7.1±1.4	8.0±2.3	7.8±2.1	0.800
Hemoglobin(g/L)	175.4±9.4	181.1±7.6	172.4±12.6	177.0±10.7	0.465
Creatinine(umol/L)	87.9±10.5	84.9±14.9	81.6±15.2	79.9±12.8	0.696
left atrium diameter (mm)	20.9±3.4	19.6±4.7	18.7±5.6	20.4±6.2	0.865

Note: The P value of one-way ANOVA comparing the four groups; n=7.

**Table 2:** Change in mean AERP before and 3 weeks after gene transfer

BCL	Time	Right atria				Left atria			
		Sham	Control	Ad-EGFP	Ad-ACE2	Sham	Control	Ad-EGFP	Ad-ACE2
350ms	baseline	118±10	109±11	108±15	111±12	115±11	107±10	108±17	113±18
	3 weeks	117±14	94±13	93±10*	106±9*	114±17	94±15	97±12^#^	111±10^#^
250ms	baseline	108±18	95±10	98±15	101±14	108±9	96±12	99±18	101±21
	3 weeks	107±19	89±12	87±13**	103±9**	109±16	85±15	83±14^##^	98±11^##^

* indicated as before vs. 3 weeks after gene transfer, P<0.05; BCL, basic cycle length.

**Table 3:** Changes in inducibility and duration of AF before and after gene transfer

		AF cases	AF inducibility (%)	Mean AF duration (s)
Sham	baseline	1	28.6	25.1±21.7
	3 weeks	3	42.9	63.6±40.2
Control	baseline	3	42.2	91.9±52.6
	3 weeks	7	100	185.0±87.4
Ad-EGFP	baseline	3	42.9	88.1±42.0
	3 weeks	7	100*	194.3±89.7**
Ad-ACE2	baseline	3	42.9	77.9±45.5
	3 weeks	4	57.1*	107.1±47.5**

AF cases, the number of dogs having spontaneous or induced AF by procedure stimulation.

* Compared with Ad-ACE2 and Ad-EGFP: P<0.05.

** Compared with Ad-ACE2 and Ad-EGFP: P<0.05.

### ACE2 expression and RAS components

**Figure 1:**
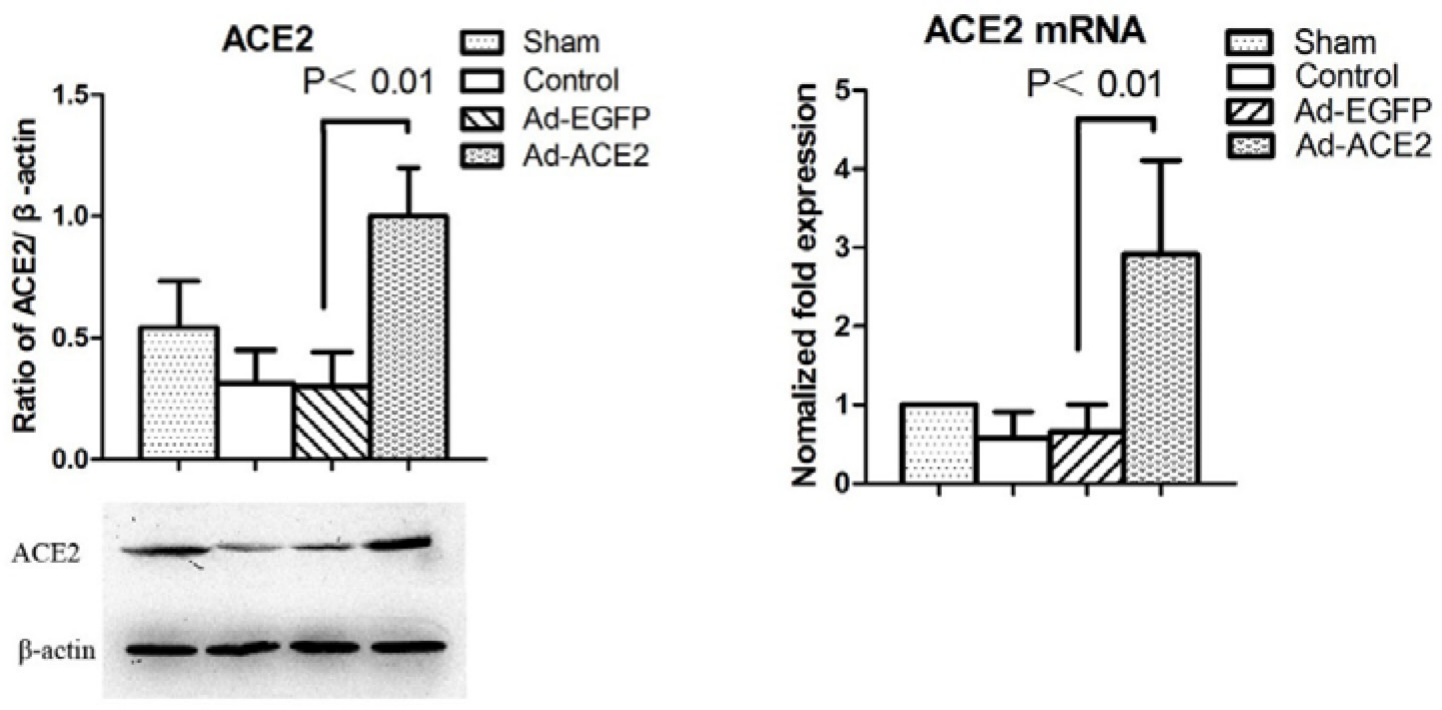
Western blot analysis of ACE2 protein levels from atrial tissues Compared to Ad-EGFP group, the protein expression level of ACE2 in Ad-ACE2 group was significantly higher. P<0.01; (n = 7).

ACE2 and RAS expression levels in each group were evaluated. ACE2 mRNA expression levels in Ad-EGFP and AF Control dogs were lower than those in Sham dogs. There was no significant difference in ACE2 mRNA expression levels between the above two experimental groups. However, ACE2 mRNA expression levels were significantly increased in Ad-ACE2 dogs compared with other dogs. Western blot analysis showed that ACE2 expression levels were significantly increased in Ad-ACE2 dogs compared with sham-operated dogs but were significantly reduced in Ad-EGFP and Control dogs compared with sham-operated dogs at 3 weeks after gene transfer (Figure 1). Compared with the sham-operated and Ad-ACE2 groups, Ang II expression levels were significantly increased in the Ad-EGFP and AF control groups. In contrast, Ang-(1-7) expression levels were lower in the Ad-EGFP and AF-control groups than in the sham-operated and Ad-ACE2 groups. Our experimental data show that rapid atrial pacing significantly reduced ACE2 and Ang-(1-7) expression levels and increased Ang II expression levels (Table 4).

**Table 4:** Enzyme-linked immunosorbent assay of Ang II and Ang-(1-7)

ng/g protein	Sham	Control	Ad-EGFP	Ad-ACE2	P
Ang II	12.9±3.0	25.4±5.7	25.6±7.4	18.2±6.0	<0.05
Ang-(1-7)	8.3±1.7	5.3±1.2	4.6±1.7	10.3±3.2	<0.01

Note: Ad-ACE2 vs. Ad-EGFP; Ang II P<0.05; Ang-(1-7) P<0.01.

### TRPM7 expression

**Figure 2:**
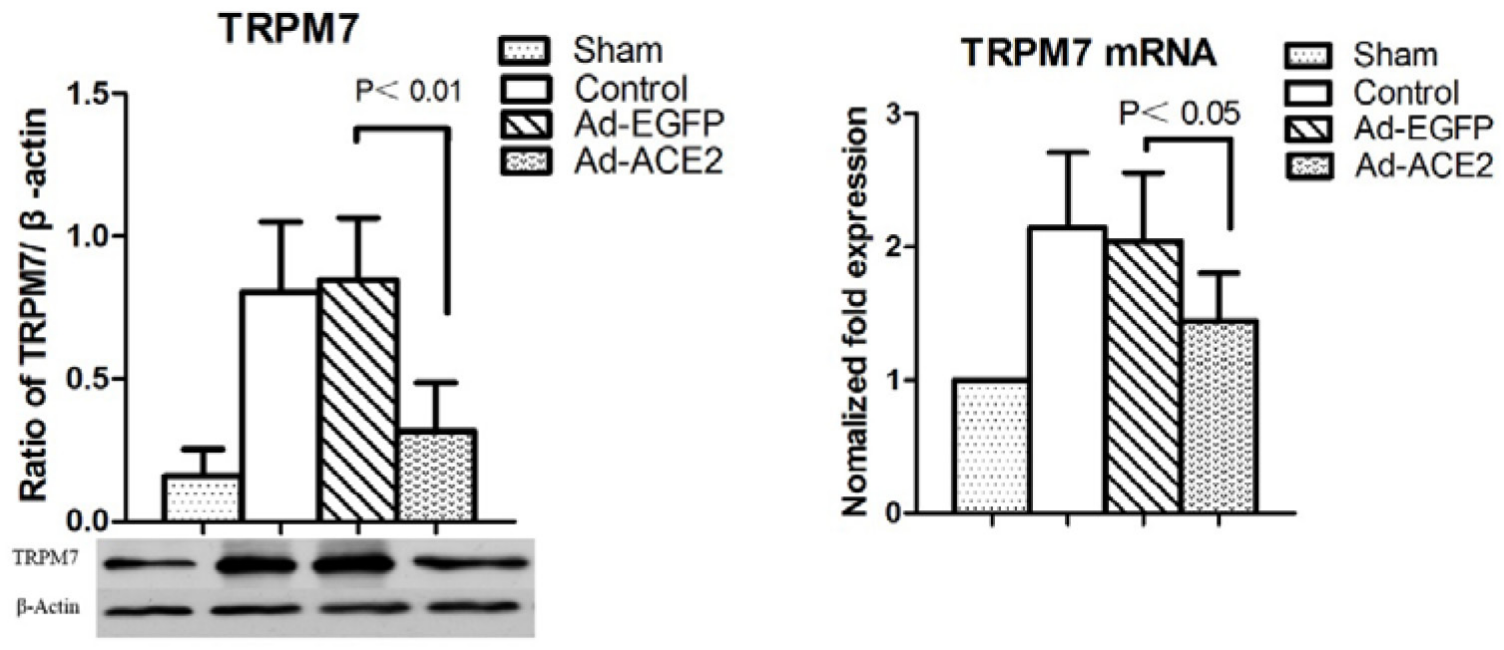
Western blot analysis of TRPM7 protein levels from atrial tissues Compared to Ad-EGFP group, the protein expression level of TRPM7 Ad-EGFP group was significantly higher; (n = 7).

TRPM7 expression was evaluated to investigate the potential mechanisms underlying the effects of ACE2 overexpression on atrial electrical remodeling. TRPM7 protein levels in the Control and Ad-EGFP groups were significantly increased compared to those in the Sham and Ad-ACE2 groups, and there was no significant difference in TRPM7 protein expression levels between the latter two groups (Figure 2).

### Cell experiments

**Figure 3:**
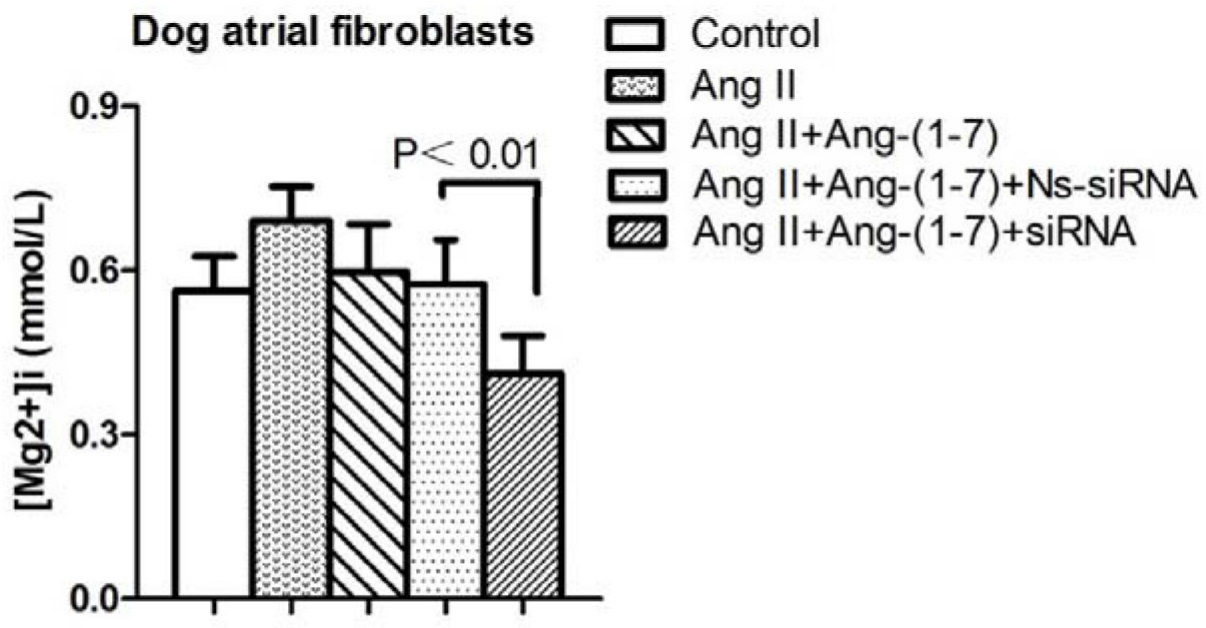
[Mg^2+^]_i_ was significantly decreased in siRNA transfected cells compared with non-silencing siRNA-transfected cells (P<0.01).

The functional significance of TRPM7 in atrial fibroblasts [Mg^2+^]_i_ regulation was assessed in cells in which *TRPM7* gene was silenced by siRNA. Basal [Mg^2+^]_i_ was significantly decreased (*P*<0.01) in siRNA transfected cells compared with control and non-silencing siRNA-transfected cells. Results were not significantly different between control and non-silencing transfected cells (Figure 3).

## DISCUSSION

### Main findings

Our study of the effects of ACE overexpression facilitated by gene transfer as a treatment for AF yielded two important findings. First, the balance between ACE and ACE2 is critical to the functional status of the RAS. Second, ACE2 may inhibit TRPM7 expression in atrial tissue during atrial electrical remodeling. To our knowledge, this study was the first to report the effects of ACE2 overexpression, which are mediated by the TRPM7 signaling pathway, on atrial electrical remodeling in experimental studies.

### Electrical remodeling and ACE2 overexpression

AF is the most important pathological condition associated with atrial electrical remodeling. Our present experimental study showed that atrial AF induction rates and AF durations were significantly increased in the AF control group and Ad-EGFP group compared with the ACE2 overexpression group and the sham-operated group. This finding is consistent with those previous studies involving various models, indicating that ACE2 overexpression induced by atrial gene transfer inhibits atrial electrical remodeling [9]. Studies have shown that AERP shortening promotes AF generation and maintenance. Both animal and human studies have demonstrated that AERP shortening which in term promoted the occurrence and persistence of AF [10]. Recent data suggested that ACE inhibitors had no protective effect against AERP shortening and did not abolish the rate adaptation in chronic atrial pacing [4]. Consistent with previous results, in our experiments, ACE2 overexpression inhibited shortening of the AERP, reduced the induction rate of AF and shortened the duration of AF [10]. Therefore, the improvement of atrial electrical remodeling by ACE2 overexpression may be the important mechanism of its effects.

### RAS components and ACE2 overexpression

The current evidence indicates that Ang II plays an important role in atrial electrical remodeling during AF [11-13]. Elevated levels of Ang II and up-regulation of AT1R in the atrial myocardium have been reported in AF patients [19]. ACE inhibitors have also been shown to block Ang II-catalyzed Ang II synthesis but not enzyme-catalyzed Ang II synthesis [14]. Thus, angiotensin II receptor blockers and ACE inhibitors do not completely inhibit RAS activation. In contrast, for the endogenous negative regulation factor, ACE2 converts Ang II to Ang-(1-7) in atrial tissue in AF, thereby exerting greater cardio-protective effects than angiotensin II receptor blockers or ACE inhibitors [15]. Decreasing ACE2 expression during AF may affect the Ang II-dependent signaling pathway. Atrial fibrosis in AF may be induced by antagonistic regulation between ACE and ACE2 expression [16]. Plasma ACE2 activity is elevated in human AF and elevated plasma ACE2 is significantly associated with more advanced LA structural remodeling [17]. Cardiac remodeling could effectively be inhibited through upregulation of the expression of the ACE2-Ang(1-7)-Mas axis and downregulation of the expression of the ACE-Ang II-AT1 axis [18]. A large body of evidence suggests that RAS activation is involved in atrial electrical remodeling [19-21]. In this study, the atrial RAS was found to be significantly activated in atrial pacing dogs, as demonstrated by increases in Ang II and decreases in Ang-(1-7) levels in these animals; however, atrial ACE2 overexpression reversed these changes. Compared to Ad-EGFP group, the protein expression level of ACE2 in Ad-ACE2 group was significantly higher. The results suggest that atrial rapid pacing gives rise to activation of RAS in atrial tissues, while ACE2 overexpression in atrial tissue leads to a shift of the RAS balance towards the protective axis.

### TRPM7 and ACE2 overexpression

Recent studies have shown that the transient receptor potential melastatin 7 (TRPM7) pathway is involved in regulating various biological activities [22-23]. Atrial electrical remodeling is an important feature of AF. Many signal transduction pathways and bioactive molecules participate in atrial electrical remodeling. TRPM7 is a non-selective cation channel that exhibits outward rectifying properties. Under physiological conditions, the inward current mediated by TRPM7 is usually very small. Intracellular Mg^2+^ can inhibit the TRPM channel. In cardiac fibroblasts, TRPM7 is the predominant Ca^2+^ channel [24-26]. Patients with AF exhibit TRPM7 current densities that are 3- to 5-fold higher than those of patients in sinus rhythm. Consistent with its upregulation in fibroblasts, TRPM7 was also upregulated in the cardiomyocytes of AF patients. Ca^2+^ inflow is much greater in AF fibroblasts than in control fibroblasts. In this study, we noted a significant increase in TRPM7 expression in the atrial tissues of AF control and Ad-EGFP dogs compared to that in the atrial tissues of Sham dogs. Cardiac fibrosis is involved in most cardiovascular diseases. Cardiac fibrosis can block conduction, create electrical heterogeneity, which is associated with the development of arrhythmia. Some researches demonstrated TRPM7 expression inhibited Ang II-induced fibrosis. Besides, knocking down TRPM7 by shRNA, they proved that TRPM7 mediated both calcium changes in cardiac fibroblasts which contribute to fibrosis progress [27]. Consistent with previous results, in our experiments, basal [Mg^2+^]_i_ was significantly decreased (*P*<0.01) in siRNA transfected cells compared with control and non-silencing siRNA-transfected cells. TRP channels are highly expressed in cardiac fibroblasts. TRPM7 has been shown to be essential in TGF mediated fibrogenesis. Thus the Ca^2+^-permeable TRP channels may serve as potential novel targets for developing anti-fibrotic drugs [28]. Previous studies demonstrated that the overexpression of ACE2 inhibited myocardial fibrosis [29]. The results of this study suggest that ACE2 reduces TRPM7 expression, leading to an increase in the atrial tissue ERP, suggesting that ACE2 overexpression mediated by TRPM7 mitigates atrial electrical remodeling. Identification of the TRPM7 channel has complemented and enriched our understanding of the molecular mechanisms underlying atrial remodeling and has provided us with a new target for AF therapies. We acknowledge that this study does not investigated the detailed molecular mechanism of the imbalance of RAS components, particularly the up-regulated or down-regulated mechanisms of those molecules, and need to be further elucidated. The potential role of TRPM7 in atrial electrical remodeling remains to be explored in future studies.

## MATERIALS AND METHODS

### Ethics statement

All the experiments were approved by the Animal Experimentation Ethics Committee of Shanghai Jiao Tong University School of Medicine.

### Adenoviral vector construction

Recombinant adenoviruses carrying the canine ACE2 gene (Ad-ACE2) or a control transgene (Ad-EGFP) were prepared by Shanghai Shenggong Biotechnologies (Shanghai, China).

### Animal model and gene transfer

Twenty-eight mongrel dogs weighing 18-32 kg were randomly divided into the following 4 groups: a Sham-operation (Sham) group, a Control group, an Ad-EGFP group and an Ad-ACE2 group (n=7 in each group). All dogs underwent baseline tests, including 12-lead ECGs, echocardiograms, routine blood tests, and serum creatinine level tests, to exclude unhealthy animals. All the dogs in the Control, Ad-EGFP and Ad-ACE2 groups were paced at 450 bpm for 14 days. Anesthesia was administered as a peritoneal injection of sodium pentobarbital (10 mg/kg). An endocardial pacing electrode was inserted through the right jugular vein [30]. The electrode was subsequently inserted into the subcutaneous pouch of the neck and connected to a pacemaker (Fudan University, Shanghai, China). The animals in the experimental groups were paced at twice the threshold current and underwent ECG monitoring every other day. The dogs in the sham-operated group did not undergo pacing [31]. After 2 weeks, all the dogs underwent thoracotomy and electrophysiological examinations, followed by epicardial gene painting. The animals subsequently underwent postoperative ECG monitoring, and their behavior was assessed daily. On day 21 after gene transfer, all the animals were subjected to electrophysiological and molecular studies.

### Electrophysiological studies

AF induction was defined as P wave disappearance, rapid atrial activity, and irregular ventricular responses, as demonstrated by atrial electrocardiography after programmed atrial stimulation (S1 to S2) and AF duration recording. The effective refractory periods (AERPs) of the left and right atrium were measured at 2 basic cycle lengths (BCLs) (250 and 350 ms). The S1-S2 interval was increased in 5 ms steps, and the AERP was determined to be the shortest S1-S2 interval leading to a transmissible atrial response.

### Molecular analysis

TRPM7 and ACE2 protein expression levels were determined by Western blotting (1:100 dilution; Santa Cruz Biotechnology, USA). Briefly, dog atrial tissue samples (∼100 mg) were homogenized in lysis buffer and then centrifuged for purification. The electrophoretically separated proteins were transferred onto a nitrocellulose membrane, which was probed with a specific antibody and then incubated with a horseradish peroxidase-conjugated secondary antibody. TRPM7 and ACE2 mRNA expression levels were determined by real-time RT-PCR. The mixture was heated at 56 °C for 2 minutes, 93 °C for 90 seconds and then 93 °C for 15 seconds and 56 °C for 30 seconds for 40 cycles. GAPDH served as an internal control. The results were quantified as Ct values. Ct was defined as the PCR cycle threshold, or the number of cycles required for the amplification products to be detected.

### Ang II and Ang-(1–7) levels by ELISA

The levels of Ang II in the atrial tissue were measured using a commercial enzyme-linked immunosorbent assay (ELISA) kit (E-EL-C0224c, Elabscience, China). In brief, the atrial tissue was mechanically homogenized on ice, using a homogenizer. The diluted tissue supernatant was placed in a 96-well goat anti-dog IgG-coated plate and incubated for 2 hours. After incubation, the plate was washed by washing buffer. The amount of Ang II was calculated using a standard curve. A similar method was used to examine the levels of Ang-(1–7) (ZK-C7231, Ziker, China).

### Fibroblasts experiment

The study approved by the Shanghai JiaoTong University School of Medicine, Animal Subject Committee for the preparation of cells. Cardiac fibroblasts were isolated from the atrial tissues of mongrel dogs. Cells from passages 1 to 2 were grown to confluence and then stimulated for 40 min in media alone or with drugs. TRPM7 silencing by small interfering RNA (siRNA) is used to confirm that TRPM7 is responsible for the currents. The selective fluorescent probes was used to measure [Mg^2+^]_i_. [Mg^2+^]_i_ responses to increasing concentrations of extracellular Mg^2+^ (0 to 5 mmol/L) were measured in cells incubated in Mg^2+^-free, Ca^2+^-containing modified Hanks’ buffer. Cells were exposed to Mg^2+^-free buffer for 15 to 20 minutes before addition of extracellular Mg^2+^.

### Statistical analysis

All quantitative data are expressed as the mean ± SD. Shapiro-Wilk’s test was used to determine whether each variable had a normal distribution. Variables were compared between groups using t tests for continuous variables and chi-square tests for categorical variables. Statistical comparisons among the groups were performed by one-way ANOVA. If significant effects were identified by ANOVA, a least significant difference (LSD) t test was used to evaluate the significance of the difference between individual mean values. A 2-tailed P<0.05 was considered statistically significant (SPSS 22, Chicago, IL).

## References

[1] Magnani JW, Rienstra M, Lin HH, Sinner MF, Lubitz SA, McManus DD, Dupuis J, Ellinor PT, Benjamin EJ (2011). Atrial fibrillation: current knowledge and future directions in epidemiology and genomics. Circulation.

[2] Kappenberger L (2013). A new look at atrial fibrillation: lessons learned from drugs, pacing, and ablation therapies. Eur Heart J.

[3] Wang X, Ye Y, Gong H, Wu J, Yuan J, Wang S, Yin P, Ding Z, Kang L, Jiang Q, Zhang W, Li Y, Ge J, Zou Y (2016). The effects of different angiotensin II type 1 receptor blockers on the regulation of the ACE-AngII-AT1 and ACE2-Ang(1-7)-Mas axes in pressure overload-induced cardiac remodeling in male mice. J Mol Cell Cardiol.

[4] Kumagai K, Nakashima H, Urata H, Gondo N, Arakawa K, Saku K (2003). Effects of angiotensin II type 1 receptor antagonist on electrical and structural remodeling in atrial fibrillation. J Am Coll Cardiol.

[5] Donoghue M, Hsieh F, Baronas E, Godbout K, Gosselin M, Stagliano N, Donovan M, Woolf B, Robison K, Jeyaseelan R, Breitbart RE, Acton S (2000). A novel angiotensin-converting enzyme-related carboxypeptidase (ACE2) converts angiotensin I to angiotensin 1-9. Circ Res.

[6] Chubanov V, Schäfer S, Ferioli S, Gudermann T (2014). Natural and synthetic modulators of the TRPM7 channel. Cells.

[7] Jiang J, Li M, Yue L (2005). Potentiation of TRPM7 inward currents by protons. J Gen Physiol.

[8] Simon F, Varela D, Cabello-Verrugio C (2013). Oxidative stress-modulated TRPM ion channels in cell dysfunction and pathological conditions in humans. Cell Signal.

[9] Fan J, Zou L, Cui K, Woo K, Du H, Chen S, Ling Z, Zhang Q, Zhang B, Lan X, Su L, Zrenner B, Yin Y (2015). Atrial overexpression of angiotensin-converting enzyme 2 improves the canine rapid atrial pacing-induced structural and electrical remodeling. Fan, ACE2 improves atrial substrate remodeling. Basic Res Cardiol.

[10] Ehrlich JR, Hohnloser SH, Nattel S (2006). Role of angiotensin system and effects of its inhibition in atrial fibrillation: clinical and experimental evidence. Eur Heart J.

[11] Boldt A, Scholl A, Garbade J, Resetar ME, Mohr FW, Gummert JF, Dhein S (2006). ACE inhibitor treatment attenuates atrial structural remodeling in patients with lone chronic atrial fibrillation. Basic Res Cardiol.

[12] Hirayama Y, Atarashi H, Kobayashi Y, Takano T (2004). Angiotensin-converting enzyme inhibitors are not effective at inhibiting further fibrous changes in the atria in patients with chronic atrial fibrillation: speculation from analysis of the time course of fibrillary wave amplitudes. Jpn Heart J.

[13] Chrysostomakis SI, Karalis IK, Simantirakis EN, Koutsopoulos AV, Mavrakis HE, Chlouverakis GI, Vardas PE (2007). Angiotensin II type 1 receptor inhibition is associated with reduced tachyarrhythmia-induced ventricular interstitial fibrosis in a goat atrial fibrillation model. Cardiovasc Drugs Ther.

[14] Kumar R, Singh VP, Baker KM (2007). The intracellular renin-angiotensin system: a new paradigm. Trends Endocrinol Metab.

[15] Backx PH, Yagil Y, Penninger JM (2000). Angiotensin-converting enzyme 2 is an essential regulator of heart function. Nature.

[16] Pan CH, Lin JL, Lai LP, Chen CL, Stephen Huang SK, Lin CS (2007). Downregulation of angiotensin converting enzyme II is associated with pacing-induced sustained atrial fibrillation. FEBS Lett.

[17] Walters TE, Kalman JM, Patel SK, Mearns M Velkoska E, Burrell LM (2016). Angiotensin converting enzyme 2 activity and human atrial fibrillation: increased plasma angiotensin converting enzyme 2 activity is associated with atrial fibrillation and more advanced left atrial structural remodelling. Europace.

[18] Wang X, Ye Y, Gong H, Wu J, Yuan J, Wang S, Yin P, Ding Z, Kang L, Jiang Q, Zhang W, Li Y, Ge J, Zou Y (2016). The effects of different angiotensin II type 1 receptor blockers on the regulation of the ACE-AngII-AT1 and ACE2-Ang(1-7)-Mas axes in pressure overload-induced cardiac remodeling in male mice. J Mol Cell Cardiol.

[19] Goette A, Lendeckel U (2004). Expression of angiotensin II receptors in human left and right atrial tissue in atrial fibrillation with and without underlying mitral valve disease. J Am Coll Cardiol.

[20] Dobrev D, Nattel S (2011). New insights into the molecular basis of atrial fibrillation: mechanistic and therapeutic implications. Cardiovasc Res.

[21] Zhao J, Xu W, Yun F, Zhao H, Li W, Gong Y, Yuan Y, Yan S, Zhang S, Ding X, Wang D, Zhang C, Dong D (2014). Chronic obstructive sleep apnea causes atrial remodeling in canines: mechanisms and implications. Basic Res Cardiol.

[22] Li M, Jiang J, Yue L (2006). Functional characterization of homo- and heteromeric channel kinases TRPM6 and TRPM7. J Gen Physiol.

[23] Antunes TT, Callera GE, He Y, Yogi A, Ryazanov AG, Ryazanova LV, Zhai A, Stewart DJ, Shrier A, Touyz RM (2016). Transient receptor potential melastatin 7 cation channel kinase: new player in angiotensin II-induced hypertension. Hypertension.

[24] Du J, Xie J, Zhang Z, Tsujikawa H, Fusco D, Silverman D, Liang B, Yue L (2010). TRPM7-mediated Ca2+ signals confer fibrogenesis in human atrial fibrillation. Circ Res.

[25] Runnels LW, Yue L, Clapham DE (2002). The TRPM7 channel is inactivated by PIP (2) hydrolysis. Nat Cell Biol.

[26] Zhang YH, Sun HY, Chen KH, Du XL, Liu B, Cheng LC, Li X, Jin MW, Li GR (2012). Evidence for functional expression of TRPM7 channels in human atrial myocytes. Basic Res Cardiol.

[27] Yu Y, Chen S, Xiao C, Jia Y, Guo J, Jiang J, Liu P (2014). TRPM7 is involved in angiotensin II induced cardiac fibrosis development by mediating calcium and magnesium influx. Cell Calcium.

[28] Yue Z, Zhang Y, Xie J, Jiang J, Yue L (2013). Transient receptor potential (TRP) channels and cardiac fibrosis. Curr Top Med Chem.

[29] Zhou T, Wang Z, Fan J, Chen S, Tan Z, Yang H, Yin Y (2015). Angiotensin-converting enzyme-2 overexpression improves atrial remodeling and function in a canine model of atrial fibrillation. J Am Heart Assoc.

[30] Gaspo R, Bosch RF, Talajic M, Nattel S (1997). Functional mechanisms underlying tachycardia-induced sustained atrial fibrillation in a chronic dog model. Circulation.

[31] Kikuchi K, McDonald AD, Sasano T, Donahue JK (2005). Targeted modification of atrial electrophysiology by homogeneous transmural atrial gene transfer. Circulation.

